# Metabolomics-Based Discovery of Molecular Signatures for Triple Negative Breast Cancer in Asian Female Population

**DOI:** 10.1038/s41598-019-57068-5

**Published:** 2020-01-15

**Authors:** Lixian Li, Xiaodong Zheng, Qi Zhou, Nathaniel Villanueva, Weiqi Nian, Xingming Liu, Tao Huan

**Affiliations:** 1 Chongqing Key Laboratory of Translational Research for Cancer Metastasis and Individualized Treatment, Chongqing University Cancer Hospital & Chongqing Cancer Institute & Chongqing Cancer Hospital, Chongqing, 400030 P.R. China; 2 Department of Chemistry, University of British Columbia, Vancouver, British Columbia V6T 1Z1 Canada; 3 Department of Breast Cancer, Chongqing University Cancer Hospital & Chongqing Cancer Institute & Chongqing Cancer Hospital, Chongqing, 400030 P.R. China

**Keywords:** Metabolomics, Breast cancer

## Abstract

Triple negative breast cancer (TNBC) is a devastating cancer disease characterized by its poor prognosis, distinct metastatic patterns, and aggressive biological behavior. Research indicates that the prevalence and presentation of TNBC varies among races, with Asian TNBC patients more commonly presenting with large invasive tumors, high node positivity, and high histologic grade. In this work, we applied ultra-high performance liquid chromatography-high resolution mass spectrometry (UHPLC-HRMS)-based metabolomics to discover metabolic signatures in Asian female TNBC patients. Serum samples from 31 TNBC patients and 31 healthy controls (CN) were involved in this study. A total of 2860 metabolic features were detected in the serum samples. Among them, 77 metabolites, whose levels were significantly different between TNBC with CN, were confirmed. Using multivariate statistical analysis, literature mining, metabolic network and pathway analysis, we performed an in-depth study of the metabolic alterations in the Asian TNBC population. In addition, we discovered a panel of metabolic signatures that are highly correlated with the 5-year survival rate of the TNBC patients. This metabolomic study provides a better understanding of the metabolic details of TNBC in the Asian population.

## Introduction

Among women, breast cancer (BC) is the most common type of cancer and also the 2^nd^ leading cause of death worldwide^1^. BC can be divided into several major subtypes based on conventional immunohistochemistry detection of hormone receptors, including human estrogen receptor (ER), progesterone receptor (PR), and human epidermal growth receptor-2 (HER2). As a highly heterogeneous disease, patients with BC have various morphological spectrum, clinical presentation, and prognostic outcomes^2^. Among the various subtypes, triple negative breast cancer (TNBC) uniquely lacks expression of all three hormone receptors and accounts for 15–20% of the BC cases. TNBC has attracted more attention compared with other BC subtypes as it is typically associated with high aggression, poor prognosis and a high risk of disease relapse within 5 years following diagnosis^3^. Women with TNBC have a high frequency of metastasis to the lung, liver and brain, and survival is generally poor. Another troubling feature associated with the disease is the disparity of presentation and survival compared with other ethnicities^4–9^. It is thus of great demand to study the molecular basis of TNBC in order to guide the development of promising drugs and therapies for treatment.

Metabolomics is an emerging technology for health science research, representing a more recent addition to the suite of “omics” tools. In particular, mass spectrometry (MS)-based metabolomic analysis enables the most comprehensive measurement of metabolites in a given biological system. It is thus a powerful analytical tool to identify metabolic biomarkers associated with disease or abnormal phenotypes for clinical applications^4^. Since metabolites are the end products of gene regulatory processes and protein activities, metabolomics has also been widely used to understand metabolic mechanisms underlying disease phenotypes in order to guide the development of better therapeutic strategies.

The prevalence of TNBC varies among different races and ethnic groups. For instance, a previous study performed in California, USA showed that Asian women have a lower lifetime risk of TNBC than white, African-American, and Hispanic counterparts^5^. Other studies indicate that TNBC among Asians shows trends of earlier age of onset and more aggressive biological behavior^6–8^. The metabolic signatures in TNBC are of critical biological importance for both mechanistic research and clinical application. However, most previous studies have been conducted in Western populations, and few in Asian populations. We believe that a comprehensive metabolomics study of TNBC among Asians would facilitate the discovery of new treatment-dependent metabolites and increase understanding of responses to treatment that occur in TNBC.

In this study, we collected serum samples from 31 TNBC patients and 31 healthy women in southwest China. We applied a UHPLC-HRMS platform for global metabolomic profiling, followed by univariate and multivariate statistical analyses to identify statistically significant metabolites in TNBC vs. CN. Our study discovered a total of 77 significantly altered metabolites, covering a wide range of metabolic classes, including lipid, amino acids, and carboxylic acids. Comparing to the reported BC metabolomics studies, we identified some consistent metabolic changes as well as some unique metabolic changes in Asian female TNBC patients. Finally, from archived prognostic data for the TNBC patients, we identified 6 metabolites that can stratify patients’ 5-year survival rate. This work presents the first metabolomics study of TNBC in Asian population, thus serving for a better mechanistic understanding of disease progression and prognosis.

## Results

### Clinical characteristics of subjects

The study was conducted in accordance with the Declaration of Helsinki, and the protocol was approved by the Ethics Committee of Chongqing Cancer Hospital. All experiments were performed in strict compliance with the requirements of the Human Ethics Procedures and Guidelines of the People’s Republic of China. Serum samples of TNBC patients were collected from hospitalized female patients with histopathologically confirmed TNBC at Chongqing Cancer Hospital (China). These patients were enrolled in this study from October 2013 to February 2015. Serum samples of the CN group were collected from the age-matched healthy participant volunteers. All serum samples were collected before any medication and surgery towards TNBC. The status of ER, PR and HER2 were negative and the cancers ranged from stages I to III. The demographic and clinical characteristics of study participants are shown in Table 1. Detailed clinical parameter for the TNBC patients are presented in Supplementary Table S1. Informed consent and ethical committee approval was obtained from every participant.

**Table 1:** Clinical features of subjects.

Subjects		TNBC	CN
Sample size		31	31
Age (years), mean (SD)		51.5 (10.2)	51.7 (10.6)
Menopause status, n	Premenopausal	9	10
	Perimenopausal	13	12
	Postmenopausal	9	9
Cancer stage, n	I	4	NA
	II	17	NA
	III	10	NA
Cancer type, n (%)	(ER−, PR−, HER2−), n (%)	100%	NA
Sample collection before or after medication		before	NA
Sample collection before or after surgery		before	NA

The table shows age and range of subjects at the time of blood sample collection.

NA: not applicable.

TNBC, Triple negative breast cancer; CN, healthy controls; ER−, negative expression of estrogen receptor; PR−, negative expression of progesterone receptor; HER2−, negative expression of human epidermal growth factor receptor.

### Metabolomics workflow

**Figure 1:**
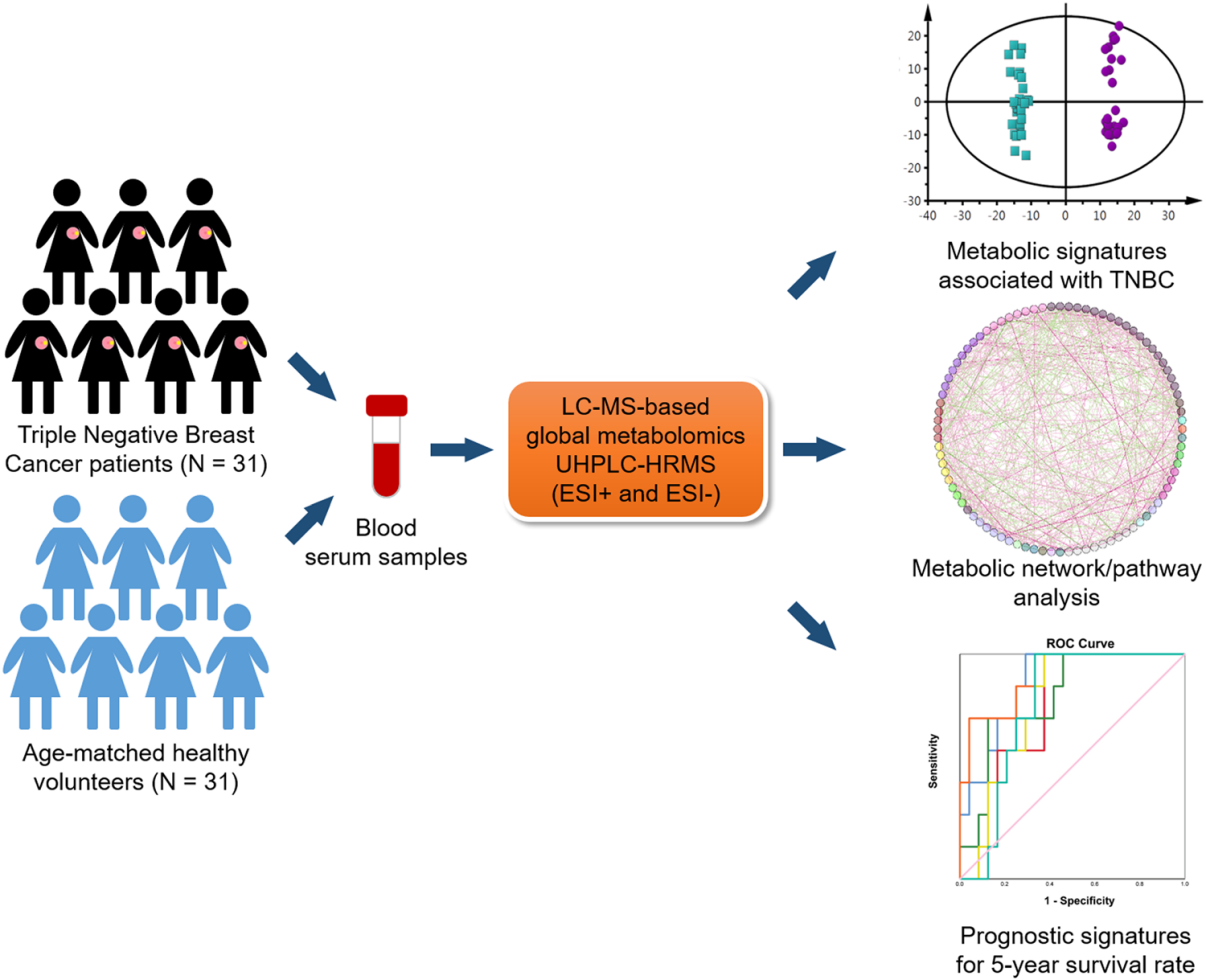
Workflow of UHPLC-HRMS-based metabolomics for metabolomic profiling and data interpretation of serum samples from TNBC and CN.

**Figure 2:**
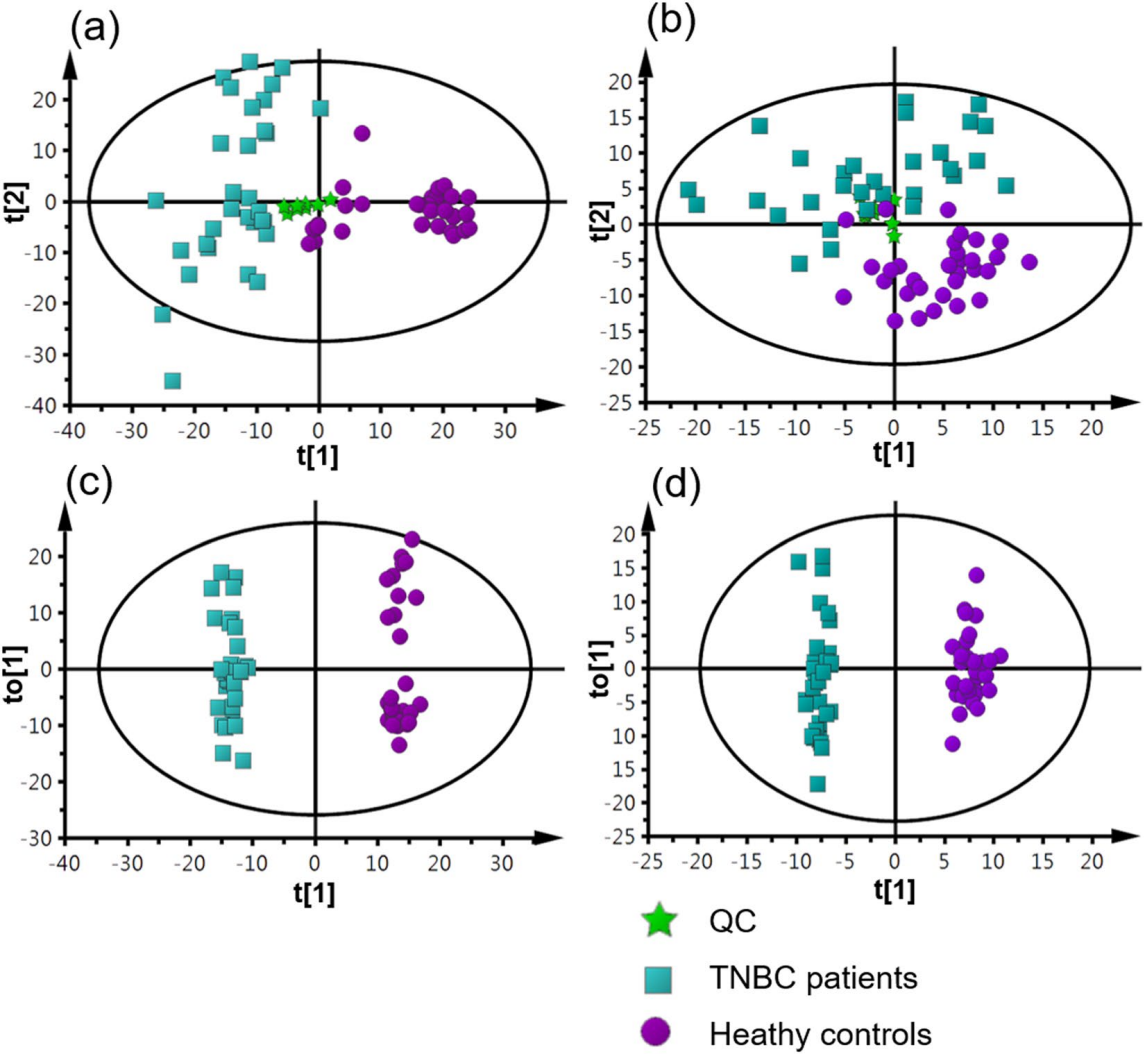
Multivariate statistical analysis results. (**a**) PCA score plot of the analysis in ESI (+) mode. (PC1 covers 11.6% of the variables; PC2 covers 6.4% of the variables). (**b**) PCA score plot of the analysis in ESI (−) mode (PC1 covers 8.9% of the variables; PC2 covers 6.1% of the variables). (**c**) OPLS-DA score plot of the analysis in ESI (+) mode (R^2^X = 0.209, R^2^Y = 0.99, Q^2^ = 0.883). (**d**) OPLS-DA score plot of the analysis in ESI (−) mode (R^2^X = 0.183, R^2^Y = 0.984, Q^2^ = 0.833).

**Figure 3:**
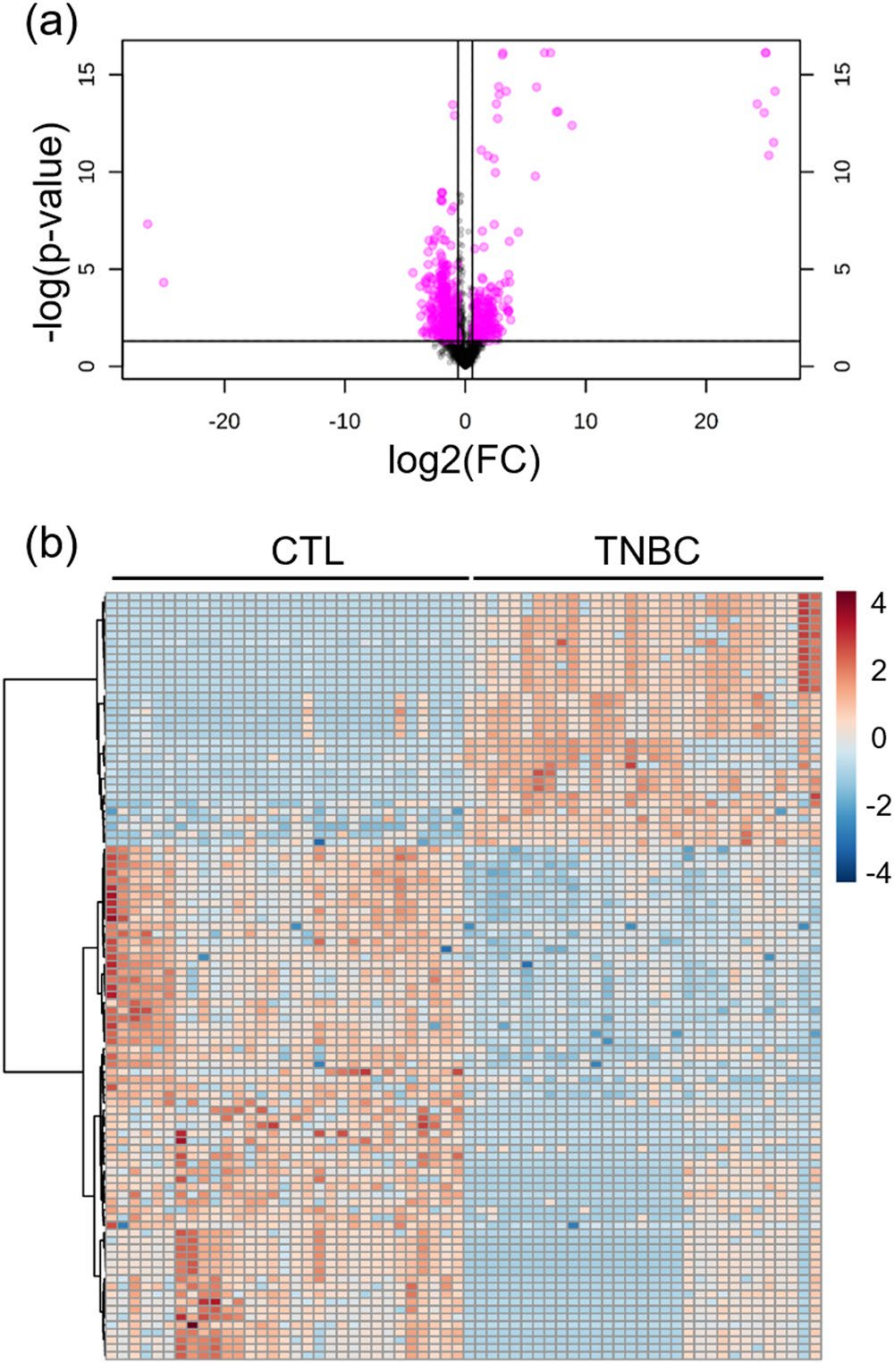
(**a**) Representative Volcano plot (fold change threshold = 1.5 and p-value (FDR adjusted) in ESI (+) mode metabolomics data: 0.05; (**b**) Representative heatmap of top 100 significant metabolites in ESI (+) mode metabolomics data.

Figure 1 shows the schematic workflow of the global metabolomics study. Serum samples were collected from 31 TNBC patients and 31 CN volunteers. Metabolites were extracted from the serum samples and analyzed using the UHPLC-HRMS platform. Both electrospray ionization positive (ESI+) and negative (ESI−) mode-based MS analyses were performed to achieve comprehensive metabolome profiling of the serum samples. After LC-MS analysis, metabolic features were extracted from each individual sample and aligned to create a metabolite-intensity table for downstream data interpretation. In total, 2860 metabolic features were consistently detected in all serum samples, including 1856 features in ESI+ mode and 1004 features in ESI− mode, respectively. The data interpretation was conducted by a suite of bioinformatic tools. Firstly, all metabolic features and their relative MS abundance were analyzed in principal component analysis (PCA) and orthogonal partial least square-discrimination analysis (OPLS-DA) to gain a global view of the metabolic differences between TNBC and CN (Fig. 2). Further, statistically significant metabolic features were extracted using the criteria of fold change ≥ 1.2 or ≤0.83 and p-value ≤ 0.05 and visualized using volcano plot and heatmap (Fig. 3). Metabolite identification was performed by comparing retention time, accurate mass, and tandem MS spectra of the metabolic features against in-house metabolite standard library as well as HMDB^9^ and METLIN^10^. Seventy-seven statistically significant metabolites were confirmed (Table 2). These metabolites were then used to construct correlation-based metabolic networking analysis and pathway enrichment analysis to better understand their biological significance. Finally, since TNBC has poor prognostic outcome, we attempted to utilize the metabolomic data to identify metabolites that are potentially correlated with the 5-year survival rate.

**Table 2:** Significantly altered metabolites in TNBC vs. healthy controls in the analysis of LC-MS positive mode and LC-MS negative mode.

Class	Subclass	Name	RT	massb	t-test	Fold change*	VIP	Mode
Benzene and substituted derivatives	Benzoic acids and derivatives	Hippuric acid	5.09	179.0580	4.E-02	0.4	1.1	neg
Carboxylic acids and derivatives	Amino acids, peptides, and analogues	Creatine	0.73	131.0692	5.E-03	1.6	1.116	Pos
		L-Leucine	1.61	131.0945	1.E-03	1.8	1.663	neg
		L-Proline	0.57	115.0634	4.E-05	1.4	1.569	Pos
		L-Threonine	0.69	119.0577	2.E-04	1.4	1.452	Pos
		L-Tyrosine	1.44	181.0736	2.E-04	1.4	1.863	neg
		L-Valine	1.06	117.0791	5.E-04	1.2	1.359	Pos
		N-Acetyl-L-Histidine	0.70	197.0802	2.E-03	1.7	1.554	neg
		Pyroglutamic acid	0.87	129.0427	3.E-02	1.4	1.119	neg
Dihydrofuranes	Furanones	L-Ascorbic acid	0.75	176.0328	1.E-05	4.3	2.137	neg
Fatty acyls	Fatty acid esters	Decanoyl-L-carnitine	7.98	315.2412	1.E-04	0.4	1.502	Pos
		Acetylcarnitine	0.75	203.1156	3.E-03	0.8	1.188	Pos
		L-Octanoylcarnitine	6.98	287.2100	5.E-04	0.5	1.357	Pos
		Propionyl-L-carnitine	2.07	217.1313	9.E-03	1.6	1.039	Pos
	Fatty acids and conjugates	Leucinic acid	5.44	132.0775	1.E-02	1.6	1.269	neg
		Oleic Acid	14.14	282.2558	4.E-02	0.8	1.092	neg
		Palmitic acid	13.98	256.2400	1.E-03	0.7	1.636	neg
	Fatty amides	Oleamide	13.42	281.2721	3.E-04	1.7	1.413	Pos
	Lineolic acids and derivatives	9(S)-HODE	12.11	296.2345	9.E-05	0.4	1.958	neg
		9(S)-HpOTrE	10.66	310.2137	7.E-03	0.4	1.391	neg
		Linoleic acid	13.50	280.2404	4.E-03	0.5	1.153	Pos/neg
		α-Linolenic Acid	12.92	277.2246	6.E-05	0.6	1.542	Pos/neg
Glycerolipids	Monoradylglycerols	MG(0:0/24:1/0:0)	14.15	440.3834	2.E-04	0.4	1.875	neg
Glycerophospholipids	Glycerophosphates	PA(15:0/0:0)	13.98	393.3095	2.E-02	0.7	1.186	neg
		PA(20:1/17:1)	10.88	714.5133	1.E-05	3.0	1.679	Pos
	Glycerophosphocholines Glycerophosphoeserines	Glycerophosphocholine	0.68	257.1025	2.E-13	0.3	2.45	Pos
		LysoPC(0:0/18:0)	11.95	523.3650	1.E-05	0.6	1.668	Pos
		LysoPC(15:0)	10.48	481.3177	2.E-08	0.3	2.025	Pos/neg
		LysoPC(16:1)	10.37	493.3174	8.E-03	1.6	1.065	Pos
		LysoPC(18:3)	10.12	517.3173	7.E-04	2.4	1.327	Pos
		LysoPC(18:4)	6.68	515.3025	9.E-06	3.4	1.681	Pos
		LysoPC(20:1)	12.40	549.3806	2.E-03	0.7	1.198	Pos
		LysoPC(20:2)	11.64	547.3646	2.E-05	0.8	1.628	Pos
		LysoPC(20:4)	10.53	543.3333	1.E-03	0.8	1.257	Pos
		LysoPC(22:5)	10.92	569.3487	2.E-04	0.5	1.435	Pos
		PC(16:0/3:0)	11.65	551.3571	6.E-06	0.7	2.213	neg
		PC(17:0/0:0)	12.18	509.3470	1.E-05	0.7	2.158	neg
		PC(17:1/18:1)	13.12	771.5719	6.E-11	3.8	2.27	Pos
		PC(20:5/0:0)	10.48	541.3151	5.E-04	1.8	1.353	Pos
		PC(O-12:0/O-1:0)	11.27	439.3042	2.E-02	0.5	1.24	neg
		PC(P-18:1/14:1)	12.66	713.5298	1.E-06	3.1	1.821	Pos
		PS(14:0/20:0)	8.59	763.5292	4.E-03	0.5	1.132	Pos
		PS(19:0/0:0)	10.31	539.3215	6.E-03	1.9	1.407	neg
		PS(20:3/21:0)	10.95	855.5941	2.E-03	0.5	1.564	neg
		PS(22:0/0:0)	11.85	581.3670	4.E-02	0.5	1.09	neg
		PS(22:0/17:2)	10.86	829.5771	1.E-02	0.5	1.301	neg
		PS(O-18:0/0:0)	10.02	511.3281	8.E-09	3.6	2.073	Pos
		PS(O-20:0/20:2)	13.55	829.6129	5.E-11	4.0	2.277	Pos
	Glycerophosphoethanolamines	LysoPE(0:0/18:1)	11.29	479.3005	7.E-03	1.2	1.387	pos/neg
		LysoPE(0:0/18:2)	10.61	477.2851	4.E-02	1.2	1.061	neg
		LysoPE(0:0/22:0)	12.75	537.3803	3.E-05	0.6	1.603	Pos
		LysoPE(0:0/22:5)	10.95	527.3004	1.E-02	1.8	1.266	neg
		LysoPE(20:1/0:0)	11.14	507.3312	2.E-04	0.5	1.874	neg
		LysoPE(22:4/0:0)	10.69	529.3158	9.E-03	0.8	1.36	neg
	Glycerophosphoglycerols	PG(10:0/10:0)	6.76	554.3248	8.E-05	3.5	1.518	Pos
		PG(16:0/0:0)	6.55	484.2806	1.E-05	3.4	1.655	Pos
		PG(18:1/0:0)	6.67	510.2983	7.E-04	2.4	1.326	Pos
		PG(22:0/18:2)	11.89	830.5975	2.E-03	3.6	1.212	Pos
		PG(22:2/22:2)	9.02	882.6263	2.E-04	0.2	1.454	Pos
	Glycerophosphoglycerophosphoglycerols	CL(18:2)	12.07	1452.9920	2.E-13	0.0	2.453	Pos
		CL(20:2)	10.88	1479.0088	4.E-03	0.1	1.134	Pos
		CL(20:3)	10.22	1467.0059	7.E-05	0.3	1.532	Pos
		CL(22:6)	9.76	1544.9748	3.E-05	0.3	1.592	Pos
	Glycerophosphoinositol phosphates	PIP(18:0/18:3)	6.30	940.5063	1.E-05	4.3	2.145	neg
	Glycerophosphoinositols	PI(18:3/22:1)	13.98	914.5961	6.E-05	3.3	1.549	Pos
		PI(22:0/16:1)	13.96	892.6115	5.E-05	2.9	1.558	Pos
		PI(22:0/18:0)	10.79	922.6567	4.E-05	0.2	1.585	Pos
Imidazopyrimidines	Purines and purine derivatives	Uric acid	1.14	168.0284	1.E-02	1.4	1.034	Pos
Indoles and derivatives	Indolyl carboxylic acids and derivatives	L-Tryptophan	4.34	204.0898	4.E-04	1.2	1.779	neg
Lactones	Gamma butyrolactones	Dehydroascorbic acid	0.75	174.0165	6.E-04	3.2	1.749	neg
Organonitrogen compounds	Quaternary ammonium salts	Phosphocholine	10.81	183.0662	9.E-14	0.4	2.471	Pos
Pyridines and derivatives	Pyridinecarboxylic acids and derivatives	Niacinamide	1.27	122.0477	3.E-03	1.6	1.172	Pos
Pyrimidine nucleotides	Pyrimidine deoxyribonucleotides	Deoxyuridine monophosphate (dUMP)	1.11	308.0422	9.E-03	1.2	1.357	neg
Steroids and steroid derivatives	Bile acids, alcohols and derivatives	Cholic acid	9.08	408.2869	6.E-03	5.8	1.422	neg
Steroids and steroid derivatives		Deoxycholic acid	10.51	392.2922	2.E-03	2.5	1.57	neg
Steroids and steroid derivatives	Hydrosteroids	corticosterone	12.92	346.2111	5.E-03	0.5	1.461	neg
Tetrapyrroles and derivatives	Bilirubins	Bilirubin	6.36	584.2638	2.E-17	0.1	2.663	Pos/neg

^*^fold change was calculated as TNBC/CN.

### Global metabolic profiles of TNBC and CN serum samples

To gain an overview of the metabolic differences between TNBC and CN, we first performed principle component analysis (PCA) of all 62 serum samples. Quality control (QC) data was also included in the PCA analysis to monitor the overall instrument robustness and stability. As shown in Fig. 2a,b, the PCA score plots clearly separate the TNBC and CN groups. In addition, the QC samples were clustered together, indicating the excellent analytical reliability of the applied metabolomics platform.

We next applied OPLS-DA, a supervised multivariate statistical analysis tool, to identify the metabolic features that contribute to the metabolic differences between TNBC and CN serum samples. The OPLS-DA score plots in Fig. 2c,d respectively show very clear separations between TNBC and CN groups in both ESI+ and ESI− modes. Metabolic features with VIP scores ≥ 1.5 in the OPLS-DA analyses were extracted and retained as significant metabolic features for downstream analysis. The quality of these OPLS-DA models were evaluated via an internal cross validation, which calculates the goodness of fit parameter (R^2^Y) and predictive ability of the model (Q^2^). In both ESI+ and ESI− analyses, we observed high R^2^Y and Q^2^ values and the differences between R^2^Y and Q^2^ are smaller than 0.2, which indicates no model overfitting. Together, these results show that there are significant and valid global metabolic differences between the serum metabolome of TNBC and CN.

### Discovery of metabolic signatures for TNBC

To identify and confirm high-confidence metabolic signatures that contribute to TNBC, we first calculated fold changes and p-values for all metabolic features in TNBC vs. CN. In total, 805 and 257 significant metabolic features (FD ≥1.2 or ≤0.83 and p-value ≤ 0.05) were found in ESI+ and ESI− analysis modes, respectively. We further refined the list of significant metabolic features using OPLS-DA VIP scores and only kept those with scores ≥1.5. The refined significant metabolic features were then searched against our in-house metabolite library to confirm the identity of 77 metabolites. By applying the chemical taxonomy in HMDB, we were able to categorize significant metabolites into 14 metabolite classes and 25 subclasses (Table 2). Of these 77 altered metabolites, 38 metabolites were upregulated in the TNBC samples and the other 39 metabolites were upregulated in the CN samples. A large portion of the dysregulated metabolites (45 out of the total 77) were in the class of glycerophospholipids. Notably, all altered amino acids were upregulated in the TNBC samples. Although directions of alteration for the significantly changed lipids are not consistent, we can observe some consistency within the subclasses of lipids. For example, all cardiolipin (CL) species are downregulated in TNBC samples. We also conducted literature mining of all 77 dysregulated metabolites to compare against other metabolomics studies of BC or TNBC. While most of the significant metabolites have been previously reported in literature, a few of them (e.g., N-acetyl-L-histidine, octanoylcarnitine) were uniquely discovered for the first time in this Asian female TNBC population. The table of detailed meta-analysis results is available in Supplementary Table S2.

### Metabolic network and pathway enrichment analysis

**Figure 4:**
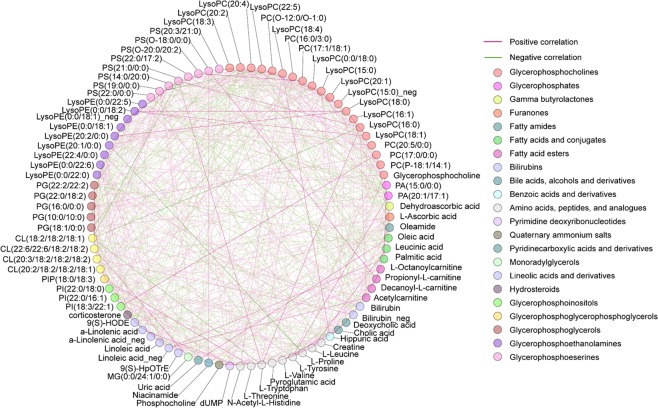
Correlation-based metabolic network analysis. Each node represents one metabolite and the edge connecting two nodes represents the correlation coefficient, with positive correlation in red and negative correlation in green.

To further understand the underlying biological meanings of these dysregulated metabolites, we performed correlation-based metabolic network analysis. Correlation-based network analysis was used to find the abundance correlation of two metabolites to infer potential biological interpretation. It provides convenient visualization of the potential biological relationships and underlying activity correlations of metabolites. The analysis results were visualized using the MetScape^11^ plugin available in Cytoscape (3.7.1)^12^. Figure 4 shows the metabolic network analysis results in circle layout, which locates all nodes in the network around a circle. In the circle network, each node represents the significantly altered metabolites and the edge between two nodes represents the correlation coefficient, with positive correlation in red and negative correlation in green. The darker color indicates the higher correlation coefficient. An edge is displayed only if the correlation between the two metabolites is ≥0.2 or ≤−0.2. The color labels of the metabolic nodes are based on their taxonomy classes identified from HMDB. From the molecular network results (Fig. 4) we can see that the dysregulated metabolites have strong correlations with each other, suggesting the important underlying biological meanings of the metabolites during the TNBC progression. Among these metabolic correlations, we identified 68 pairs of metabolic correlation with coefficient ≥0.4 or ≤−0.4 (Supplementary Table S3).

**Figure 5:**
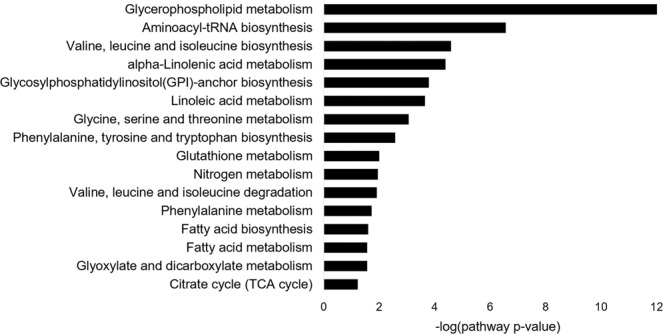
Metabolic pathway analysis. Predicted metabolic pathways with p-value ≤ 0.05 are listed.

To further understand the metabolic changes on the pathway level, we performed pathway enrichment analysis in MetaboAnalyst (https://www.metaboanalyst.ca). A total of 16 metabolic pathways were predicted with pathway significance p-value ≤ 0.05 Fig. 5, Supplementary Table S4) with the top three significantly altered metabolic pathways being glycerophospholipid metabolism, aminoacyl-tRNA biosynthesis, and valine, leucine and isoleucine biosynthesis.

### Metabolic signatures correlated with 5-year survival rate in TNBC patients

**Figure 6:**
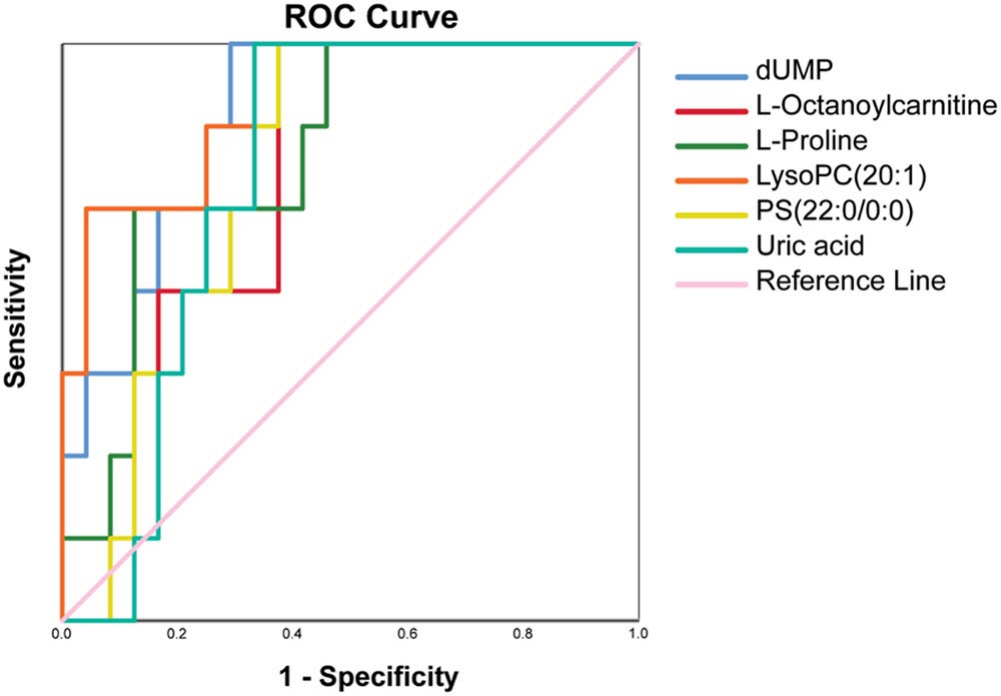
ROC curves for dUMP, L-octaoylcarnitine, L-proline, LysoPC (20:1), PS (22:0/0:0), and uric acid.

TNBC is known to have poor prognostic outcomes; therefore metabolites correlated with prognostic outcomes at an early stage could guide further therapeutic intervention. In this study, we focused on the 5-year survival rate, which is an important statistic that reflects cancer progress and treatment success. Out of the 31 TNBC cases, 7 patients were deceased within 5 years of treatment and the other 24 patients survived 5 years following treatment. After log transformation and auto-scaling of the dataset, we performed Receiver Operating Characteristic (ROC) analysis of all significant metabolic features using IBM SPSS statistics. We successfully discovered 6 metabolites with Area Under Curve (AUC) values larger than 0.75 (Fig. 6). These metabolites are dUMP, L-octanoylcarnitine, L-proline, lysoPC (22:1), PS (22:0/0:0), and uric acid (Table 3).

## Discussion

The overall goal of this study was to delineate the unique serum metabolic signature for TNBC in the Asian female population. Upon applying a state-of-the-art metabolomics platform on serum samples from 31 well-classified TNBC patients and 31 CN volunteers, we were able to detect a large number of significantly changed metabolites that are associated with TNBC and further confirmed 78 of them. Through comprehensive data interpretation, we acquired a better understanding of the metabolic features in Asian female TNBC patients. Finally, further statistical analysis suggested that six of the metabolites were well-correlated with the 5-year TNBC survival rate. To our present knowledge, this is the first metabolomics study of TNBC in the Asian female population.

One unique feature of this study is that all of the serum samples were collected after disease diagnosis and before drug treatment. Therefore, identified metabolic changes were the direct reflection of altered cancer metabolism rather than metabolic perturbations caused by therapeutic intervention. In our study, many of these metabolic changes are consistent with previous BC or TNBC studies of blood, tissue samples of human population or cell culture-based studies. For instance, the dysregulation of glycerophospholipids, including phosphatidylcholine (PC), phosphatidylethanolamine (PE), phosphatidylserine (PS), phosphatidylglycerol (PG), lysophosphatidylcholine (lysoPC), lysophosphatidylethanolamine (lysoPE), monoradylglycerolipid (MG), phosphatidic acid (PA), cardiolipin (CL) have been reported in many other types BC or TNBC studies^13–15^. We noted several metabolites as novel in our study, including N-acetyl-L-histidine, phosphatidylinositol (PIP), phosphatidylinositol (PI), and dehydroascorbic acid. These metabolic signatures could be sourced from the unique genetic, lifestyle, and other environmental factors, leading to the unique TNBC phenotype in Asian females, such as being diagnosed at a younger age of 40–50 and more aggressive disease behavior^16^. Therefore, these unique metabolic signatures could potentially be used to further investigate the intriguing mechanism of race-specific TNBC phenotypes and disease outcome.

Further metabolic network analysis (Fig. 4) suggested that the alterations of various metabolic concentrations in the serum of TNBC patients were not independent of each other. Instead, correlation-based metabolic network analysis suggested inherited correlations among the dysregulated metabolites. The most significant correlations between these metabolites are indicative of particular phenotypes or biological aberrations. While the dysregulated metabolic metabolites and metabolic pathways have been well investigated in disease phenotypes, the association between metabolites in disease conditions is not well studied. In this work, we provided a comprehensive correlation-based metabolic network for all dysregulated metabolites (Fig. 4, Supplementary Table S3). It thus provides rich information for further investigation to understand the underlying metabolic mechanisms for TNBC progression.

Beyond the alteration of individual metabolites, further pathway enrichment analysis systematically predicted the alteration of metabolic pathways (Fig. 5, Supplementary Table S4). The systems level alteration of metabolites in glycerophospholipid metabolism pathway is consistent with metabolomics studies of BC in other populations^14,17^. The altered lipid concentrations are important to cell membrane modeling and inflammation and have been reported to be associated with advanced metastatic BC in cell lines^18^, serum^19^, plasma^20^, and tissue^21,22^.

The consistent upregulation of amino acids in this study suggested that the pathway of aminoacyl-tRNA biosynthesis was disrupted. Aminoacyl-tRNA biosynthesis, an essential process for protein synthesis and cell viability^23^, conjugates amino acids to tRNA and delivers amino acids for incorporation into polypeptide chains. Cancer proliferation requires large amounts of biomass to sustain tumor growth, and the elevated concentration of amino acids in the blood is likely due to the increased demand for protein synthesis in TNBC patients.

Another unique feature of this study is that all participating patients were recruited in 2013–2015 and consistent follow-ups on prognosis outcomes were maintained. For instance, 5-year survival and cancer metastasis status are available for all patients. Determination of prognosis is the most immediate challenge in patient management and is critically important for the design of the most appropriate cancer therapy to improve survival. The development of traditional prognostic factors (*e.g*., lymph node metastasis, tumor size, and tumor grade) and molecular prognostic biomarkers (*e.g*., uPA/PAl1, Oncotype DX, and MammaPrint) has demonstrated great success for BC prognosis^24^. Apart from the aforementioned tissue biopsy-based analysis, the use of “liquid biopsy” from a blood sample has become an appealing expectation as it provides a non-invasive and easily accessible tool for patient stratification. From that perspective, mi-RNA, circulating tumor cells, and circulating tumor DNA-based assays have been developed and have shown promising predictive power. Since metabolites are the downstream product of gene and protein activity, metabolites can potentially be used as prognostic biomarkers.

Using this prognostic information, six metabolites (Table 3), including dUMP, L-octanoylcarnitine, L-proline, lysoPC (22:1), PS (22:0/0:0), and uric acid, were discovered as highly associated with 5-year survival rate. dUMP is the precursor of dTMP, which is necessary for DNA synthesis and repair. The conversion of dUMP to dTMP is catalyzed by thymidylate synthase. A previous study in colon cancer demonstrated that high level of thymidylate synthase is associated with lymph node metastasis and more advanced stages^25^. Similarly, L-octanoylcarnitine, an important metabolite in carnitine metabolism, has been shown to be a prognostic marker to differentiate between prostate cancer and benign prostatic hyperplasia^26^. L-proline is a non-essential amino acid in humans. Proline can feed the TCA cycle through the urea cycle and is oxidized by proline dehydrogenase to form reactive oxygen species (ROS). The potential application of proline as the biomarkers for BC diagnosis and prognosis has been previously suggested, though the mechanisms leading to its perturbance are unclear^27^. LysoPC is an abundant extracellular lipid that stimulates cell proliferation. Previous studies have shown that lysoPC is not only significantly altered in BC^28^ but can also stimulate cancer cell migration and early tumor recurrence^23,29^. PS is an essential component of human cells and presents mainly on the inner leaflet of the cell membrane; however, the oxidative stress in BC cells can cause the exposure of PS. We believe that the higher concentration of PS (22:0/0:0) observed in the study is correlated with higher metastasis and death rate of TNBC patients. Finally, serum uric acid has long been known as a significant risk factor for excessive cancer risk, recurrence, and mortality^30^

**Table 3:** Six metabolites associated with the 5-year survival rate of TNBC patients.

Metabolite name	ROC area	Std. Errors	Sensitivity	Specificity	Asymptotic 95% Confidence Interval	
					Lower Bound	Upper Bound
dUMP	0.875	0.063	85.7%	75.0%	0.751	0.999
L-Octanoylcarnitine	0.774	0.085	85.7%	62.5%	0.608	0.940
L-Proline	0.810	0.084	85.7%	58.3%	0.645	0.974
LysoPC(22:1)	0.805	0.060	71.4%	95.8%	0.778	1.000
PS(22:0/0:0)	0.770	0.081	85.7%	66.7%	0.621	0.939
Uric acid	0.774	0.081	71.4%	75.0%	0.615	0.933

. It is therefore expected that the elevated concentration of uric acid (or called hyperuricemia) in TNBC patients could be a good indicator of poor 5-year survival rate.

Among the limitations of the present study is the relatively small to medium sample size. This is attributed to the relatively low percentage of TNBC in the overall incidence of BC. Future work is needed in a more targeted approach to validate the discovery using a larger cohorts, ultimately including samples from population-wide case-control studies.

In summary, we presented a global metabolomics study of TNBC patients in the Asian population. We observed significantly altered metabolites in TNBC serum samples and developed a metabolite-based prognostic biomarker panels for the prediction of 5-year survival rate of TNBC. The application of information-rich analytical methods provides insights into understanding metabolic signatures that are associated with TNBC. We expect that our study will facilitate the development of better treatment strategies to combat TNBC within the Asian population. It may also open up new possibilities for the development of personalized medicine for TNBC patients.

## Methods

### Serum sample collection

Before treatment of BC, 3–5 ml blood samples were drawn from 31 TNBC patients and 31 healthy controls, respectively. The clinical characteristics of subjects were summarized in Table 1 and detailed clinical parameters were presented in Supplementary Table 1. Blood samples were incubated at room temperature for 30 min to allow the blood to clot. To purify serum samples, the clotted blood was centrifuged for 5 min at 3000 r/min. The upper serum layer was extracted and stored in −80 °C until needed.

### Metabolite extraction

Prior to LC-MS analysis, 400 μL methanol was added to 100 μL serum sample in a 1.5 ml Eppendorf tube and vortex-mixed for 30 s to precipitate serum proteins and extract serum metabolites. The sample mixture was centrifuged at 12000 rpm for 15 min at 4 °C. 200 μL of the supernatant was transferred to LC vials for LC-MS analysis. Samples were kept at 4 °C throughout the analysis^31^.

### Metabolomic profiling by UHPLC-QTOF MS

LC-MS analysis was carried out using an Agilent 1290 Infinity ultrahigh performance liquid chromatography system coupled to an Agilent 6530 UHD and Accurate-Mass QTOF MS. An Agilent Zorbax C18 column (100 mm × 2.1 mm, 1.8 μm particle size) was used for LC separation. Mobile phase A was water in 0.1% fomic acid (FA) and mobile phase B was ACN in 0.1% FA. The flow rate was set as t = 0 min, 5% B; t = 1 min, 5% B; t = 6 min, 20% B; t = 9 min, 50% B; t = 13 min, 95% B; t = 15 min, 95% B. Additional 10 min post gradient run at 5% B was performed to re-equilibrium the column for the next analysis. The LC flow rate was 0.35 mL min^−1^. The column was maintained at 40 °C. Experimental sample injection order was randomized and each sample was injected 4 μL.

The Agilent QTOF MS was equipped with an electrospray ionization (ESI) source operating in either positive or negative ion mode. The ESI+ with the spray voltage set at 4,000 V, Sampling cone 3,500 V. Nitrogen was used as nebulizer gas, and nebulizer gas was delivered at a flow rate of 50 L h^−1^ with a source temperature of 100 °C. Extraction cone 4 V. Desolvation gas (nitrogen) was heated to 350 °C and delivered at a flow rate of 600 L h^−1^.

The negative ion mode with the capillary voltage set at 3 500 V, Sampling cone 5 000 V, Source temperature 100 °C, Desolvation temperature 300 °C, Cone gas flow 50 L/h, Desolvation gas flow 700 L h^−1^, Extraction cone 4 V, Scan time 0.03 s, Inter scan time) 0.02 s. Masses were acquired from m/z 50 to 1,000 with a scan time of 0.03 s and inter scan delay of 0.02 s over a 15 min analysis time.

The MS was operated in full scan mode. Data dependent LC-tandem MS was performed on the pooled sample to collect fragmentation spectra for metabolite identification. Leucine encephalin (100 ng/ml) was used as the lock mass ([M^+^H]^+^, m/z 556.2771 in ESI (+) and [M^−^H]^−^, m/z 554.2615 in ESI (−)) for internal mass calibration.

QC sample was prepared by taking 10 µL of each individual sample and pooled them together. QC served as “technical replicates” for the study and was analyzed in between every 8 sample injection.

### Data processing, statistical analysis and metabolite identification

Agilent Mass Profiler software was used to extract metabolic features from the LC-MS data and generate a metabolite-intensity table containing the retention time, accurate mass and intensities of all metabolites found in the samples.

Prior to any statistical analysis, data transformation and data scaling were performed on metabolic features using log transformation and auto scaling (mean-centered and divided by the standard deviation of each metabolic features) Multivariate statistical analysis, including principle component analysis (PCA) and orthogonal signal correction partial least squares discrimination analysis (OPLS-DA) was performed on SIMCA-P (version 13.0). Univariate statistical analysis, including volcano plot, fold changes, and t-test statistics were performed using MetaboAnalyst 4.0 (https://www.metaboanalyst.ca/)^32^.

Metabolite identification was performed by matching experimental tandem MS spectra, retention time, and accurate mass of the metabolic features against in-house standard tandem MS spectra library as well as spectral databases such as METLIN and HMDB.

### Correlation-based metabolic network analysis and metabolic pathway analysis

Correlation-based metabolic networking analysis was performed using the MetScape 3 plugin available on Cytoscape 3.7. After log transformation and auto scaling, the confirmed significantly changed metabolites and their MS signal intensities were used to calculate the Pearson’s correlation coefficient and debiased squared partial correlation results using the CorrelationCalculator available on the MetScape website (http://metscape.ncibi.org/calculator.html). The correlation file was then uploaded onto the MetScape plugin on Cytoscape to visualize the correlation network.

Metabolic pathway analysis was performed using the pathway analysis function available on MetaboAnalyst 4.0 (https://www.metaboanalyst.ca/)^32^. The 77 confirmed significant metabolites were entered as the input list. A hypergeometric test was used to evaluate the pathway significance. Homo sapiens (KEGG) was used as the pathway library for prediction analysis. The hypergeometric test and relative-betweenness centrality were selected for over representation analysis and pathway topology analysis, respectively.

### Discovery and evaluation the performance of prognostic biomarkers

Diagnostic performance was evaluated on the IBM SPSS statistics 25 platform. After log transformation and auto scaling, the confirmed significantly changed metabolites and their MS signal intensities were used in SPSS for ROC analysis.

### Ethical approval and informed consent

The study was conducted in accordance with the Declaration of Helsinki, and the protocol was approved by the Ethics Committee of Chongqing Cancer Hospital. All experiments were performed in strict compliance with the requirements of the Human Ethics Procedures and Guidelines of the People’s Republic of China.

## Supplementary information

Supplementary Information.
