# Supplementary material for: Metabolomics-Based Discovery of Molecular Signatures for Triple Negative Breast Cancer in Asian Female Population
Source: Sci Rep. 2020 Jan 15;10:370. doi: 10.1038/s41598-019-57068-5 (PMC6962155; doi:10.1038/s41598-019-57068-5)
Supplement: Supplementary file 1 — Supplementary Information. [file 41598_2019_57068_MOESM1_ESM.pdf]

Supplementary Information

Metabolomics-Based Discovery of Molecular Signatures for Triple Negative Breast Cancer in Asian Female Population

Lixian Li [1,2*†], Xiaodong Zheng[3†], Qi Zhou[1], Nathaniel Villanueva[2], Weiqi Nian [1*], Xingming Liu[1], Tao Huan[2*]

[1] Chongqing Key Laboratory of Translational Research for Cancer Metastasis and Individualized Treatment, Chongqing University Cancer Hospital & Chongqing Cancer Institute & Chongqing Cancer Hospital, Chongqing, 400030, P.R. China.

[2] Department of Chemistry, University of British Columbia, Vancouver, British Columbia, Canada V6T 1Z1

[3] Department of Breast Cancer, Chongqing University Cancer Hospital & Chongqing Cancer Institute & Chongqing Cancer Hospital, Chongqing, 400030, P.R. China.

[†] Lixian Li and Xiaodong Zheng have contributed equally to this work

* Corresponding: lilixian2010@yahoo.com (L. L.)

nwqone@126.com (W. N.)

thuan@chem.ubc.ca (T. H.), +1 (604) 8224891

Table of contents

Supplemental Table S1. Detailed clinical parameters of the 31 TNBC subjects………3

Supplemental Table S2. 77 significant metabolites and their literature information….4

Supplemental Table S3. Metabolites with strong correlations (correlation coefficient ≥ 0.4 or ≤ -0.4)…………………………………………………………………13

Supplemental Table S4. Significantly dysregulated metabolic pathways (p-value ≤ 0.05) predicted from metabolic enrichment analysis in MetaboAnalyst 4.0…………16

Supplemental Table S1. Deatiled clinical parameters of the 31 TNBC subjects.

Subjects NO.	gender	Age (years)	Tumor Stage	Menopause status	Tumor size (mm)	5-year survival status(Yes or No)
1	female	48	III	Premenopausal	19*21	No
2	female	49	I	Perimenopausal	14*18	Yes
3	female	46	I	Premenopausal	11*18	Yes
4	female	65	III	Postmenopausal	16*64	No
5	female	49	II	Perimenopausal	22*26	Yes
6	female	67	III	Postmenopausal	51*61	No
7	female	77	II	Postmenopausal	24*30	Yes
8	female	48	II	Perimenopausal	17*20	Yes
9	female	50	II	Perimenopausal	17*18	No
10	female	47	II	Premenopausal	18*26	No
11	female	47	III	Perimenopausal	12*14	Yes
12	female	59	I	Postmenopausal	14*23	Yes
13	female	48	II	Perimenopausal	22*25	Yes
14	female	61	II	Postmenopausal	12*24	Yes
15	female	67	II	Postmenopausal	5*21	Yes
16	female	39	II	Premenopausal	18*24	Yes
17	female	52	II	Perimenopausal	30*29	Yes
18	female	41	II	Perimenopausal	27*14	Yes
19	female	36	II	Premenopausal	30*26	Yes
20	female	51	II	Perimenopausal	20*24	Yes
21	female	37	III	Premenopausal	18*57	Yes
22	female	50	III	Perimenopausal	23*35	Yes
23	female	64	II	Postmenopausal	23*17	Yes
24	female	51	I	Perimenopausal	20*15	Yes
25	female	42	II	Premenopausal	25*19	Yes
26	female	30	II	Premenopausal	13*29	Yes
27	female	48	III	Premenopausal	18*24	No
28	female	64	III	Postmenopausal	15*21	Yes
29	female	51	III	Perimenopausal	26*73	No
30	female	51	II	Perimenopausal	30*25	Yes
31	female	60	III	Postmenopausal	19*35	Yes

5-year survival status (Yes/No). Yes: is still survival after 5-years follow-up; No: is descended during the 5-years follow-up.

The expression of ER, PR and HER2 of 31 TNBC subjects are all negative expression.

Supplemental Table S2. 77 significant metabolites and their literature information

Subclass	Name (Biological Sample)	Serum		Plasma		Tissue		Urine or saliva		Cell lines	
		BC	TNBC	BC	TNBC	BC	TNBC	BC	TNBC	BC	TNBC
Benzoic acids and derivatives	Hippuric acid	[1]		[2]				[3] urine<br>[4] urine			
Amino acids, peptides, and analogues	Creatine	[5, 6]				[7]<br>TPBC<br>[8]<br>[9-11]	[7] [8]	[12] urine<br>[13] urine		[14]<br>[15]<br>Luminal A	[15]
	L-Leucine							[13]urine<br>[4] urine		[14][15]<br>[16][17]	[14] [15]
	L-Proline	[18]		[19]<br>[20]	[21]	[8, 22]	[8]<br>[22]			[14][15]<br>[17] [23]	[14]<br>[15]
	L-Threonine	[24]<br>[25]<br>[1]		[26]<br>[20]		[8, 22]	[8, 22]	[4] urine		[14] [15]<br>[27]	[14]
	L-Tyrosine	[18]		[20]<br>[19]	[19]	[8, 22]	[8, 22]	[28] salivary		[14] [15]	[14] [15]<br>[29]
	L-Valine	[30]		[20, 31]		[8]<br>[22]	[8]<br>[22]	[4] urine<br>[13]urine<br>[30] urine<br>[32] salivary<br>[28] salivary		[14][15]	[14][15]

	N-Acetyl-L-Histidine										
	Pyroglutamic acid										
Furanones	L-Ascorbic acid			[33]						[34]	
Fatty acid esters	Decanoyl-L-carnitine			[35]							
	Acetylcarnitine			[20]							
	L-Octanoylcarnitine										
	Propionyl-L-carnitine										
Fatty acids and conjugates	Leucinic acid										
	Oleic Acid			[35]						[36]	
	Palmitic acid			[35] [37]			[22]				
Fatty amides	Oleamide										
Lineolic acids and derivatives	9(S)-HODE	[38]									
	9(S)-HpOTrE										
	Linoleic acid	[25]		[35]							
	α-Linolenic Acid									[39]	
Monoradylglycerols	MG(0:0/24:1/0:0)										
Glycerophosphates	PA(15:0/0:0)	[30]		[35]				[4] urine			
	PA(20:1/17:1)										
Glycerophosphocholines	Glycerophosphocholine			[40]		[7] [41] [42]	[7]	[28] salivary			
	LysoPC(0:0/18:0)	[30]		[20] [35]				[45] salivary		[17]	
	LysoPC(15:0)										

	LysoPC(16:1)			[40] [43] [44]							
	LysoPC(18:3)										
	LysoPC(18:4)										
	LysoPC(20:1)										
	LysoPC(20:2)										
	LysoPC(20:4)										
	LysoPC(22:5)										
	PC(16:0/3:0)			[40] [43] [44]				[45] salivary			
	PC(17:0/0:0)										
	PC(17:1/18:1)										
	PC(20:5/0:0)										
	PC(O-12:0/O-1:0)										
	PC(P-18:1/14:1)										
Glycerophosphoeserines	PS(14:0/20:0)	[30]						[45] salivary			
	PS(19:0/0:0)										
	PS(20:3/21:0)										
	PS(22:0/0:0)										
	PS(22:0/17:2)										
	PS(O-18:0/0:0)										
	PS(O-20:0/20:2)										
Glycerophosphoethanolamines	LysoPE(0:0/18:1)			[20]				[45] salivary		[17]	
	LysoPE(0:0/18:2)										
	LysoPE(0:0/22:0)										
	LysoPE(0:0/22:5)										
	LysoPE(20:1/0:0)										

	LysoPE(22:4/0:0)										
Glycerophosphoglycerols	PG(10:0/10:0)			[35]				[4] urine			
	PG(16:0/0:0)										
	PG(18:1/0:0)										
	PG(22:0/18:2)										
	PG(22:2/22:2)										
Glycerophosphoglycerophosphoglycerols	CL(18:2)										
	CL(20:2)										
	CL(20:3)										
	CL(22:6)										
Glycerophosphoinositol phosphates	PIP(18:0/18:3)										
Glycerophosphoinositols	PI(18:3/22:1)										
	PI(22:0/16:1)										
	PI(22:0/18:0)										
Purines and purine derivatives	Uric acid			[46]		[22]	[22]			[47]	
Indolyl carboxylic acids and derivatives	L-Tryptophan					[22]	[22]	[13, 48]<br>[10] urine			
Gamma butyrolactones	Dehydroascorbic acid										
Quaternary ammonium salts	Phosphocholine	[49]				[7]<br>[22]	[7]<br>[22]			[50] [51]<br>[52]	[53]
Pyridinecarboxylic	Niacinamide										

acids and derivatives											
Pyrimidine deoxyribonucleotides	Deoxyuridine monophosphate (dUMP)										
Bile acids, alcohols and derivatives	Cholic acid									[54]	
	Deoxycholic acid			[55]							
Hydrosteroids	corticosterone									[56]	
Bilirubins	Bilirubin	[57]									

BC including subgroups of luminal A, luminal B 1, luminal B2, HER2 overexpression.

TNBC is ER−, PR−, HER2−.

Breast cancer subgroups：luminal A (ER + /PR + , HER2- −, and Ki-67 < 20%), luminal B 1 or Lu- minal B HER2 − (ER + /PR ±, HER2 −, and Ki-67≥20%), luminal B2 or Luminal B HER2+ (ER+ /PR±, and HER2+, also named TPBC Triple positive breast cancer), HER2 overexpression (ER−, PR−, HER2 +) and TN type (ER−, PR−, HER2−).

References of literature mining:

1. Huang J-H, Fu L, Li B et al. Distinguishing the serum metabolite profiles differences in breast cancer by gas chromatography mass spectrometry and random forest method. RSC Adv. 2015; 5: 58952-58958.
2. Chen Y, Zhong M, Halmurat U et al. Metabonomics Studies on Plasma and Urine of Patients with Breast Cancer Using NMR Spectroscopy. Science & Technology Review 2014; 32: 58-63.
3. Mutsaers HAM, Heuvel LP, Van Den, Ringens LHJ et al. Uremic toxins inhibit transport by breast cancer resistance protein and multidrug resistance protein 4 at clinically relevant concentrations. Plos One 2011; 6: e18438.
4. Cala M, Aldana J, Sánchez J et al. Urinary metabolite and lipid alterations in Colombian Hispanic women with breast cancer: A pilot study. Journal of Pharmaceutical & Biomedical Analysis 2018; 152: 234.
5. Pan H, Xia K, Zhou W et al. Low Serum Creatine Kinase Levels in Breast Cancer Patients: A Case-Control Study. Plos One 2013; 8: e62112.
6. Scambia G, Spina MA, Turriziani A et al. Elevated serum levels of creatine kinase BB in breast cancer. European Journal of Gynaecological Oncology 1985; 6: 45.

7. Cao MD, Lamichhane S, Lundgren S et al. Metabolic characterization of triple negative breast cancer. Bmc Cancer 2014; 14: 1-12.

8. Kanaan YM, Sampey BP, Beyene D et al. Metabolic Profile of Triple-negative Breast Cancer in African-American Women Reveals Potential Biomarkers of Aggressive Disease. Cancer Genomics Proteomics 2014; 11: 279-294.

9. Bathen TF, Jensen LR, Sitter B et al. MR-determined metabolic phenotype of breast cancer in prediction of lymphatic spread, grade, and hormone status. Breast Cancer Research & Treatment 2007; 104: 181-189.

10. Bathen TF, Geurts B, Sitter B et al. Feasibility of MR Metabolomics for Immediate Analysis of Resection Margins during Breast Cancer Surgery. Plos One 2013; 8: e61578.

11. Giskeødegård GF, Grinde MT, Sitter B et al. Multivariate Modeling and Prediction of Breast Cancer Prognostic Factors Using MR Metabolomics. Journal of Proteome Research 2010; 9: 972-979.

12. Yu L, Jiang C, Huang S et al. Analysis of urinary metabolites for breast cancer patients receiving chemotherapy by CE-MS coupled with on-line concentration. Clinical Biochemistry 2013; 46: 1065-1073.

13. Slupsky CM, Steed H, Wells TH et al. Urine metabolite analysis offers potential early diagnosis of ovarian and breast cancers. Clinical Cancer Research An Official Journal of the American Association for Cancer Research 2010; 16: 5835.

14. Winnike JH, Stewart DA, Pathmasiri WW et al. Stable Isotope-Resolved Metabolomic Differences between Hormone-Responsive and Triple-Negative Breast Cancer Cell Lines. Int J Breast Cancer 2018; 2018: 2063540.

15. Stewart DA, Winnike JH, Mcritchie SL et al. Metabolomics Analysis of Hormone-Responsive and Triple-Negative Breast Cancer Cell Responses to Paclitaxel Identify Key Metabolic Differences. Journal of Proteome Research 2016; 15: 3225.

16. Shennan D, Thomson J, If, Travers M, Barber M. L-Leucine transport in human breast cancer cells (MCF-7 and MDA-MB-231): kinetics, regulation by estrogen and molecular identity of the transporter. Biochim Biophys Acta 2004; 1664: 206-216.

17. Peng ZX, Wang Y, Gu X et al. Metabolic transformation of breast cancer in a MCF-7 xenograft mouse model and inhibitory effect of volatile oil fromSaussurea lappaDecne treatment. Metabolomics 2015; 11: 636-656.

18. Asiago VM, Alvarado LZ, Shanaiah N et al. Early detection of recurrent breast cancer using metabolite profiling. Cancer Research 2016; 70: 8309.

19. Shen J, Yan L, Liu S et al. Plasma Metabolomic Profiles in Breast Cancer Patients and Healthy Controls: By Race and Tumor Receptor Subtypes. Translational Oncology 2013; 6: 757-765.

20. Yong F, Xin Z, Tian-Song X et al. Human plasma metabolomics for identifying differential metabolites and predicting molecular subtypes of breast cancer. Oncotarget 2016; 7: 9925-9938.

21. Kanaan YM, Sampey BP, Beyene D et al. Metabolic profile of triple-negative breast cancer in African-American women reveals potential biomarkers of aggressive disease. Cancer Genomics Proteomics 2014; 11: 279-294.

22. Yamashita Y, Nishiumi S, Kono S et al. Differences in elongation of very long chain fatty acids and fatty acid metabolism between triple-negative and hormone receptor-positive breast cancer. BMC Cancer 2017; 17: 589.

23. Lane AN, Tan J, Wang Y et al. Probing the metabolic phenotype of breast cancer cells by multiple tracer Stable Isotope Resolved Metabolomics. Metabolic Engineering 2017; 43: S109671761730040X.

24. Loch CM, Ramirez AB, Liu Y et al. Use of high density antibody arrays to validate and discover cancer serum biomarkers. Molecular Oncology 2008; 1: 313-320.

25. Wei S, Liu L, Zhang J et al. Metabolomics approach for predicting response to neoadjuvant chemotherapy for breast cancer. Molecular Oncology 2013; 7: 297-307.

26. Dougan MM, Li Y, Chu LW et al. Metabolomic profiles in breast cancer:a pilot case-control study in the breast cancer family registry. Bmc Cancer 2018; 18: 532.

27. Sun L, He X, Yang C et al. MicroRNAs and Energy Metabolism in Cancer Cells. 2014.

28. Sugimoto M, Wong DT, Hirayama A et al. Capillary electrophoresis mass spectrometry-based saliva metabolomics identified oral, breast and pancreatic cancer-specific profiles. Metabolomics 2010; 6: 78-95.

29. Goode G, Gunda V, Chaika NV et al. Correction: MUC1 facilitates metabolomic reprogramming in triple-negative breast cancer. Plos One 2017; 12: e0176820.

30. Wu J, Yang R, Zhang L et al. Metabolomics research on potential role for 9-cis-retinoic acid in breast cancer progression. Cancer Science 2018; 109: 2315-2326.

31. Lécuyer L, Victor AB, Deschasaux M et al. NMR metabolomic signatures reveal predictive plasma metabolites associated with long-term risk of developing breast cancer. International Journal of Epidemiology 2017.

32. Zhang L, Xiao H, Karlan S et al. Discovery and Preclinical Validation of Salivary Transcriptomic and Proteomic Biomarkers for the Non-Invasive Detection of Breast Cancer. Plos One 2010; 5: e15573.

33. Wu K, Helzlsouer KJ, Alberg AJ et al. A prospective study of plasma ascorbic acid concentrations and breast cancer (United States). Cancer Causes & Control 2000; 11: 279-283.

34. Kim KN, Pie JE, Park JH et al. Retinoic acid and ascorbic acid act synergistically in inhibiting human breast cancer cell proliferation. Journal of Nutritional Biochemistry 2006; 17: 454-462.

35. Cala MP, Aldana J, Medina J et al. Multiplatform plasma metabolic and lipid fingerprinting of breast cancer: A pilot control-case study in Colombian Hispanic women. Plos One 2018; 13: e0190958.

36. Menéndez JA, Del MBM, Montero S et al. Effects of gamma-linolenic acid and oleic acid on paclitaxel cytotoxicity in human breast cancer cells. European Journal of Cancer 2001; 37: 402-413.

37. Jasbi P, Wang D, Cheng SL et al. Breast cancer detection using targeted plasma metabolomics. J Chromatogr B Analyt Technol Biomed Life Sci 2019; 1105: 26-37.
38. Dowling P, Henry M, Meleady P et al. Metabolomic and proteomic analysis of breast cancer patient samples suggests that glutamate and 12-HETE in combination with CA15-3 may be useful biomarkers reflecting tumour burden. Metabolomics 2015; 11: 620-635.
39. Mason-Ennis JK, Lemay-Nedjelski LP, Wiggins AKA, Thompson LU. Exploration of mechanisms of α-linolenic acid in reducing the growth of oestrogen receptor positive breast cancer cells (MCF-7). Journal of Functional Foods 2016; 24: 513-519.
40. Xie G, Zhou B, Zhao A et al. Lowered circulating aspartate is a metabolic feature of human breast cancer. Oncotarget 2015; 6: 33369-33381.
41. Giske?Deg?Rd GF, Grinde MT, Beathe S et al. Multivariate modeling and prediction of breast cancer prognostic factors using MR metabolomics. Journal of Proteome Research 2010; 9: 972-979.
42. Serkova NJ, Niemann CU. Pattern recognition and biomarker validation using quantitative H-NMR-based metabolomics. Expert Review of Molecular Diagnostics 2006; 6: 717-731.
43. Yuan B, Schafferer S, Tang Q et al. A plasma metabolite panel as biomarkers for early primary breast cancer detection. Int J Cancer 2019; 144: 2833-2842.
44. Corona G, Polesel J, Fratino L et al. Metabolomics biomarkers of frailty in elderly breast cancer patients. J Cell Physiol 2014; 229: 898-902.
45. Zhong L, Cheng F, Lu X et al. Untargeted saliva metabonomics study of breast cancer based on ultra performance liquid chromatography coupled to mass spectrometry with HILIC and RPLC separations. Talanta 2016; 158: 351-360.
46. Jové M, Collado R, Quiles JL et al. A plasma metabolomic signature discloses human breast cancer. Oncotarget 2017; 8: 19522-19533.
47. Fini MA, David OW, Beata K et al. Migratory activity of human breast cancer cells is modulated by differential expression of xanthine oxidoreductase. Journal of Cellular Biochemistry 2010; 105: 1008-1026.
48. Chen Y, Zhang R, Song Y et al. RRLC-MS/MS-based metabonomics combined with in-depth analysis of metabolic correlation network: finding potential biomarkers for breast cancer. Analyst 2009; 134: 2003-2011.
49. Hammad LA, Wu G, Saleh MM et al. Elevated levels of hydroxylated phosphocholine lipids in the blood serum of breast cancer patients. Rapid Communications in Mass Spectrometry Rcm 2010; 23: 863-876.
50. Rachel KB, Dalia S, Dalia RS et al. Metabolic markers of breast cancer: enhanced choline metabolism and reduced choline-ether-phospholipid synthesis. Cancer Research 2002; 62: 1966-1970.
51. Singer S, ., Souza K, ., Thilly WG. Pyruvate utilization, phosphocholine and adenosine triphosphate (ATP) are markers of human breast tumor progression: a 31P- and 13C-nuclear magnetic resonance (NMR) spectroscopy study. Cancer Research 1995; 55: 5140-5145.
52. Aboagye EO, Bhujwalla ZM. Malignant transformation alters membrane choline phospholipid metabolism of human mammary epithelial cells. Cancer Research 1999;

59: 80.

53. Kamila C, Lu J, Greenwood TR et al. Mass spectrometry images acylcarnitines, phosphatidylcholines, and sphingomyelin in MDA-MB-231 breast tumor models. Journal of Lipid Research 2013; 54: 333-344.

54. Baker PR, Wilton JC, Jones CE et al. Bile acids influence the growth, oestrogen receptor and oestrogen-regulated proteins of MCF-7 human breast cancer cells. Br J Cancer 1992; 65: 566-572.

55. Costarelli V, Sanders TA. Plasma deoxycholic acid concentration is elevated in postmenopausal women with newly diagnosed breast cancer. European Journal of Clinical Nutrition 2002; 56: 925-927.

56. Shpilberg Y, Connor MK, Riddell MC. The direct and indirect effects of corticosterone and primary adipose tissue on MCF7 breast cancer cell cycle progression. Hormone Molecular Biology & Clinical Investigation 2015; 22: 91-100.

57. Liu X, Meng QH, Ye Y et al. Prognostic significance of pretreatment serum levels of albumin, LDH and total bilirubin in patients with non-metastatic breast cancer. Carcinogenesis 2015; 36: 243-248.

Supplemental Table S3. Metabolites with strong correlations (correlation coefficient ≥ 0.4 or ≤ -0.4)

Metabolite 1	Metabolite 2	Correlation Coefficient	p-value
Decanoyl-L-carnitine	L-Octanoylcarnitine	1	2.3E-08
LysoPC(18:0)	LysoPC(18:1)	1	1.9E-03
LysoPE(0:0/18:1)	LysoPE(0:0/18:1)_neg	1	1.4E-05
LysoPC(18:0)	LysoPE(0:0/18:2)	1	8.0E-04
LysoPC(18:0)	LysoPE(20:2/0:0)	1	4.4E-04
LysoPE(0:0/18:2)	LysoPE(20:2/0:0)	1	1.2E-04
Linoleic acid_neg	Palmitic acid	1	1.6E-06
PC(17:1/18:1)	PS(O-20:0/20:2)	1	2.7E-10
L-Proline	Pyroglutamic acid	1	2.2E-05
Propionyl-L-carnitine	PS(O-18:0/0:0)	0.967	1.3E-05
L-Tryptophan	LysoPC(16:0)	0.935	1.3E-02
LysoPE(0:0/18:1)_neg	PS(21:0/0:0)	0.928	3.1E-03
Leucinic acid	L-Leucine	0.925	3.4E-05
L-Threonine	α-Linolenic Acid_neg	0.848	4.4E-05
Oleic Acid	Uric acid	0.846	4.6E-05
Deoxyuridine monophosphate (dUMP)	Pyroglutamic acid	0.829	1.7E-03
LysoPC(16:0)	PS(21:0/0:0)	0.803	2.9E-02
L-Tryptophan	PS(21:0/0:0)	0.78	3.1E-02
Acetylcarnitine	Linoleic acid_neg	0.76	9.1E-03
LysoPC(18:1)	LysoPE(0:0/18:2)	0.71	2.9E-02
PC(17:0/0:0)	PI(18:3/22:1)	0.662	5.8E-04
Cholic acid	L-Valine	0.57	2.2E-03

Hippuric acid	LysoPC(18:4)	0.564	2.7E-03
N-Acetyl-L-Histidine	PIP(18:0/18:3)	0.554	2.7E-03
L-Tyrosine	PA(20:1/17:1)	0.545	3.6E-03
Acetylcarnitine	Decanoyl-L-carnitine	0.539	4.1E-02
PA(15:0/0:0)	Palmitic acid	0.537	1.4E-02
Deoxycholic acid	Hippuric acid	0.531	4.4E-03
CL(22:6/22:6/18:2/18:2)	PG(22:2/22:2)	0.521	7.4E-03
CL(22:6/22:6/18:2/18:2)	MG(0:0/24:1/0:0)	0.51	6.4E-03
Niacinamide	PS(O-18:0/0:0)	0.501	1.3E-02
Leucinic acid	Niacinamide	0.496	1.7E-02
L-Threonine	LysoPC(18:4)	0.493	1.1E-02
LysoPE(0:0/18:2)	PC(20:5/0:0)	0.487	8.0E-02
Acetylcarnitine	LysoPC(16:0)	0.483	1.4E-01
LysoPE(0:0/22:0)	LysoPE(0:0/22:5)	0.482	7.4E-03
LysoPC(15:0)_neg	LysoPC(22:5)	0.477	1.3E-02
PC(17:0/0:0)	PG(16:0/0:0)	0.477	9.1E-03
Bilirubin_neg	CL(20:3/18:2/18:2/18:2)	0.475	1.1E-02
L-Tryptophan	Pyroglutamic acid	0.452	1.7E-01
PC(16:0/3:0)	α -Linolenic Acid_neg	0.451	1.7E-02
L-Tyrosine	Propionyl-L-carnitine	0.446	2.3E-02
Creatine	L-Valine	0.442	1.6E-02
L-Tyrosine	PC(P-18:1/14:1)	0.439	1.6E-02
Dehydroascorbic acid	L-Ascorbic acid	0.438	1.5E-02
Creatine	L-Tyrosine	0.435	1.8E-02
LysoPC(15:0)_neg	PC(16:0/3:0)	0.425	2.2E-02

L-Threonine	α -Linolenic Acid	0.425	2.5E-02
Deoxyuridine monophosphate (dUMP)	L-Proline	0.421	8.9E-02
Niacinamide	PI(22:0/16:1)	0.421	2.2E-02
LysoPC(16:0)	LysoPC(18:1)	0.419	2.0E-01
PG(22:2/22:2)	PS(14:0/20:0)	0.414	3.2E-02
L-Tryptophan	PC(17:1/18:1)	0.409	2.2E-01
9(S)-HpOTrE	LysoPE(0:0/22:6)	0.408	2.2E-02
CL(20:3/18:2/18:2/18:2)	PS(14:0/20:0)	0.405	3.1E-02
Cholic acid	LysoPE(0:0/22:0)	0.404	2.7E-02
PC(16:0/3:0)	PI(18:3/22:1)	0.401	3.0E-02
9(S)-HODE	CL(20:2/18:2/18:2/18:1)	0.4	2.4E-02
LysoPC(0:0/18:0)	PS(14:0/20:0)	-0.413	2.4E-02
LysoPC(16:1)	Oleamide	-0.434	1.8E-02
Creatine	Glycerophosphocholine	-0.439	1.5E-02
PG(18:1/0:0)	PS(20:3/21:0)	-0.44	1.4E-02
Deoxyuridine monophosphate (dUMP)	MG(0:0/24:1/0:0)	-0.465	2.1E-02
L-Proline	LysoPE(20:1/0:0)	-0.545	1.6E-02
LysoPC(22:5)	PS(19:0/0:0)	-0.557	3.2E-03
CL(18:2/18:2/18:1)	PS(21:0/0:0)	-0.781	3.6E-02
CL(18:2/18:2/18:1)	L-Tryptophan	-0.941	1.3E-02
CL(18:2/18:2/18:1)	LysoPC(16:0)	-1	4.4E-03

Supplemental Table S4. Significantly dysregulated metabolic pathways (p-value ≤ 0.05) predicted from metabolic enrichment analysis in MetaboAnalyst 4.0.

**Pathway Name**	**P value**
Glycerophospholipid metabolism	1.28E-13
Aminoacyl-tRNA biosynthesis	2.74E-07
Valine, leucine and isoleucine biosynthesis	2.54E-05
alpha-Linolenic acid metabolism	4.02E-05
Glycosylphosphatidylinositol(GPI)-anchor biosynthesis	1.63E-04
Linoleic acid metabolism	2.21E-04
Glycine, serine and threonine metabolism	8.64E-04
Phenylalanine, tyrosine and tryptophan biosynthesis	2.65E-03
Glutathione metabolism	1.01E-02
Nitrogen metabolism	1.11E-02
Valine, leucine and isoleucine degradation	1.22E-02
Phenylalanine metabolism	1.87E-02
Fatty acid biosynthesis	2.53E-02
Glyoxylate and dicarboxylate metabolism	2.71E-02
Fatty acid metabolism	2.71E-02
Citrate cycle (TCA cycle)	5.86E-02
